# Supplementary material for: Data-driven multiscale dynamical framework to control a pandemic evolution with non-pharmaceutical interventions
Source: PLoS One. 2023 Jan 17;18(1):e0278882. doi: 10.1371/journal.pone.0278882 (PMC9844884; doi:10.1371/journal.pone.0278882)
Supplement: S1 File — (ZIP) [file pone.0278882.s001.zip › SupportingInformation/coronaSI.pdf]

Supporting Information

A Supplementary Figures

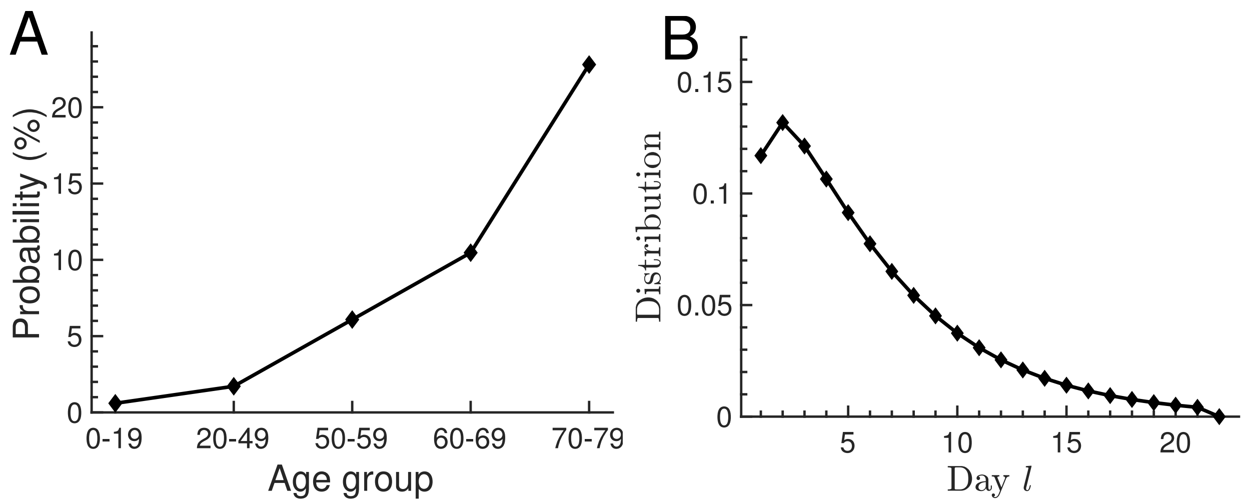

Figure S1: **Probability and time distribution for a symptomatic person to become hospitalised.** The mathematical expression for the fitting distribution is given by Eq. 26 with $l_{max} = 22$ days. (A) The fitting results for the age stratified probability that a symptomatic person becomes hospitalised are 0.6%, 1.7%, 6.1%, 10.5% and 22.8%. (B) Fitting result for the distribution that specifies at which day l after showing symptoms the hospitalisation occurs. We assumed the same distribution for all age groups. The mean number of days is 5.9.

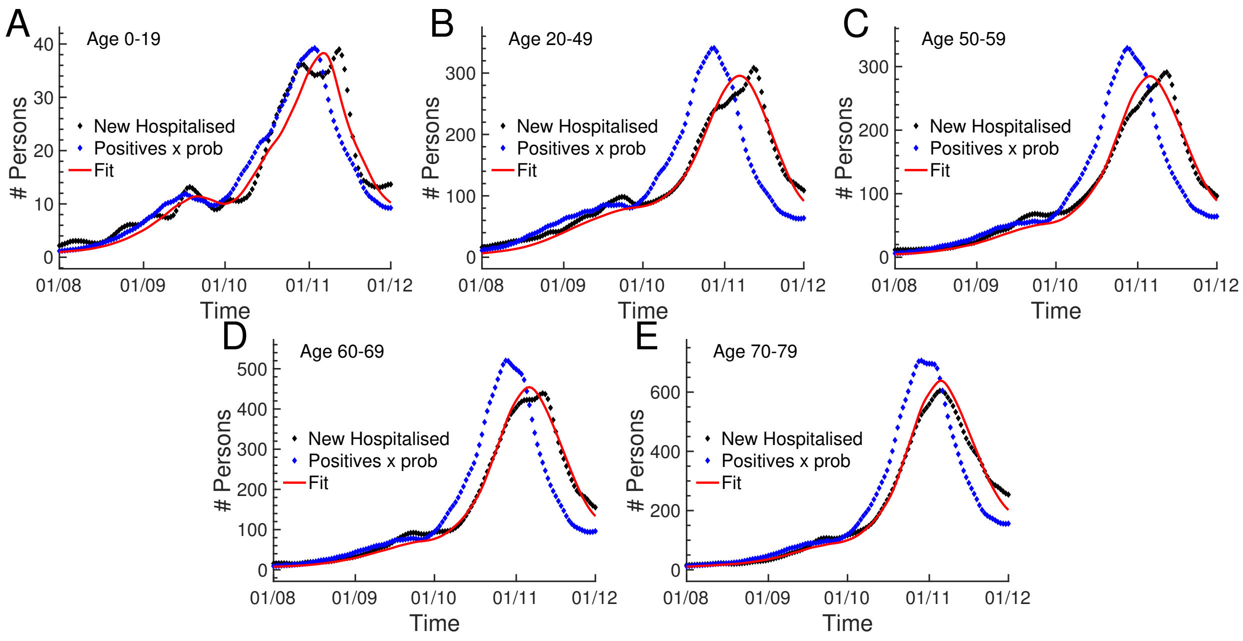

Figure S2: **Correlation between positive testings and new hospitalisations.** For each age group we fitted the probability that a positive tested person becomes hospitalised using the data for the number of new hospitalisations (black dots) and the data for the number of positive tested persons (blue dots; the data is multiplied by the fitted probability). The fitting result is shown in red. The mathematical expression for the fitting distribution is given by Eq. 26 with $l_{max} = 21$ days. The fitted probabilities are 0.6%, 1.5%, 4.5%, 10.5% and 22.8%.

.

B Modelling framework

B.1 Model structure

The parameters, variables and probability distributions of the model are:

- k: Index for the age stratified groups.
- $1 \leq j \leq 5$: Index of infection compartments.
  j=1 asymptomatic; j=2 symptomatic; j=3 in hospital; j=4 in ICU; j=5 in hospital after ICU.
- Time scales.
  n: Time measured as number of days since 01/01/2020.
  l: Number of days since an infected person joined its current compartment j.
  h: Vector that specifies the previous infection history. For example, the infection history of a hospitalised person that is currently in compartment $j = 5$ since l days comprises the number of days l_1 that this person has been asymptomatic, the number of days l_2 that it has been symptomatic, the number of hospitalisation days l_3 before ICU, and the number of days l_4 in ICU, such that $h = (l_1, l_2, l_3, l_4)$. In this case the number of days since infection are $m = l_1 + l_2 + l_3 + l_4 + l$. The vector h is updated each time the infection compartment is changed. However, in the current implementation we consider only the time scales n and l.
- $S_k(n)$: Number of susceptible persons in group k at time n.
- $\Phi_k(n)$: Number of new infected in group k at time n.
- $I_{k,h,j,l}(n)$: Number of infected at time n belonging to age group k, infection history h, current compartment j at day l after joining this compartment . For $j = 1$ the infection history is fully determined by l, and here we can omit the index h.
- $p_{k,h,j,l;j'}(n)$: Probability to transition from compartment j at day l to compartment $j' \neq j$ for an infected in age group k with history h. The index n indicates that these probabilities might change over time due to social measures or modified hospital procedures.
- $p_{k,h,j,l;rec}(n)$: Probability to recover from compartment j.
- $p_{k,h,j,l;dec}(n)$: Probability to die in compartment j.
- $p_{k,h,j,l;j}(n) = 1 - \sum_{j' \neq j} p_{k,h,j,l;j'}(n) - p_{k,h,j,l;rec}(n) - p_{k,h,j,l;dec}(n)$: Probability to remain in compartment j.
- $R_{k,h,j,l}(n) = p_{k,h,j,l;rec}(n) I_{k,h,j,l}(n)$: Number of person that recover at time n from compartment j.
- $D_{k,h,j,l}(n) = p_{k,h,j,l;dec}(n) I_{k,h,j,l}(n)$: Number of persons that die in compartment j at time n. We consider that infected only die in ICU ($j = 4$).

The recurrence relations for the time evolution are

$$\begin{aligned}
S_k(n+1) &= S_k(n) - \Phi_k(n) \quad \text{(reduction of susceptible)} \\
I_{k,1,1}(n+1) &= \Phi_k(n) \quad \text{(new infections)} \\
I_{k,h,j,l+1}(n+1) &= p_{k,h,j,l;j}(n) I_{k,h,j,l}(n) \quad \text{(evolution within the same infection compartment)} \\
I_{k,h',j,1}(n+1) &= \sum_{j' \neq j,h,l} p_{k,h,j',l;j}(n) I_{k,h,j',l}(n) \quad \text{(switching to a new infection compartment)}
\end{aligned} \tag{1}$$

For each age group k we have the conservation equation

$$\begin{aligned} S_k(n+1) + \sum_{j,h,l} I_{k,h,m,l}(n+1) &= S_k(n) + \sum_{j,h,l} I_{k,h,j,l}(n) - \sum_{j,h,l} \left(R_{k,h,j,l}(n) + D_{k,h,j,l}(n)\right) \\ &= S_k(1) + \sum_{j,h,l} I_{k,j,h,l}(1) - \sum_{n' \leq n,j,h,l} \big(R_{k,h,j,l}(n') + D_{k,h,j,l}(n')\big) \\ &= N_k - \sum_{n' \leq n,j,h,l} \big(R_{k,h,j,l}(n') + D_{k,h,j,l}(n')\big) \end{aligned} \tag{2}$$

where N_k is the initial population. If $n = 1$ corresponds to the very beginning of the pandemic, we have $N_k = S_k(1) + \Phi_k(0)$, where $\Phi_k(0)$ is the initial seed of new infections.

B.2 New infections

We assume that the number of new infections $\Phi_k(n)$ in group k at day n is proportional to the daily number of contacts $\mathcal{C}_{k;k',j,m,l}(n)$ that susceptible persons in group k make with infected persons from groups k' in compartment j,

$$\Phi_k(n) = \sum_{k',h,j,l} \mathcal{I}_{k;k',h,j,l}(n)\mathcal{C}_{k;k',h,j,l}(n)\,. \tag{3}$$

The proportionality constants are the infectivity parameters $\mathcal{I}_{k;k',h,j,l}(n)$. We simplify and assume that as soon as the infection manifests itself by showing symptoms (switching to compartment $j \geq 2$) an infected person takes precautions to avoid infecting others, in which case new infections only occur in interactions with asymptomatic persons ($j = 1$). With this assumption we have (note that $h = l$ for $j = 1$, and we omit h in the following)

$$\mathcal{I}_{k;k',j,l}(n) = \beta(n)\hat{\beta}_{k;k'}\xi_{k',l}\delta_{j,1}\,, \tag{4}$$

where $\delta_{i,j}$ is the Kronecker delta. The matrix $\hat{\beta}_{k;k'}$ models group specificities in the infection dynamics (we choose a normalization $\hat{\beta}_{k;k'} \leq 1$; the matrix $\hat{\beta}_{k;k'}$ does not have to be symmetric). For example, with $\hat{\beta}_{k;1} < 1$ and $\hat{\beta}_{1;k'} < 1$ we consider the possibility that age group 1 is less infectious and less susceptible to infections. The parameters $\xi_{k',l}$ describe how the contagiousness evolves as a function of the time l after infection (we normalize and use $\xi_{k',l} \leq 1$). The time dependent infectivity parameter $\beta(n)$ accounts for changes that equally affect all groups, e.g. the introduction of masks. The initial value of $\beta(n)$ depends on the normalisations of the other parameters. Moreover, if the affinity for mask wearing would be different among age groups, one would have to consider a time dependent matrix $\hat{\beta}_{k;k'}(n)$.

To approximate the interactions between susceptible and asymptomatic persons we use

$$\mathcal{C}_{k;k',1,l}(n) \approx c_{k;k'}(n)\frac{S_k(n)}{N_k}\frac{I_{k',1,l}(n)}{N_{k'}} \tag{5}$$

where $c_{k;k'}(n)$ is the total daily number of contacts between groups k and k', and $\frac{S_k(n)}{N_k}\frac{I_{k',1,l}(n)}{N_{k'}}$ is the fraction of these contacts that correspond to encounters between susceptible and asymptomatic. The contact matrix $c_{k;k'}(n)$ is symmetric by definition, $c_{k;k'}(n) = c_{k';k}(n)$. The matrix is time dependent

to account for social measures. Before lockdown we use [1]

$$c_{k;k'} = \begin{pmatrix} & gp_1 & gp_2 & gp_3 & gp_4 & gp_5 \\ gp_1 & 0.2656 & 0.1182 & 0.0202 & 0.0129 & 0.0044 \\ gp_2 & 0.1182 & 0.3196 & 0.0745 & 0.0505 & 0.0147 \\ gp_3 & 0.0202 & 0.0745 & 0.0394 & 0.0225 & 0.0056 \\ gp_4 & 0.0129 & 0.0505 & 0.0225 & 0.0323 & 0.0128 \\ gp_5 & 0.0044 & 0.0147 & 0.0056 & 0.0128 & 0.0065 \end{pmatrix} . \tag{6}$$

The contact matrix in Eq. 6 is normalized such that the total number of contacts is one, $\sum_{k,k' \geq k} c_{k;k'} = 1$.

Finally, with Eqs. 3-5 the number of new infections at day n generated in group k by infectious persons is

$$\Phi_k(n) = I_{k,1,1}(n+1) = \frac{S_k(n)}{N_k} \sum_{k'} \beta(n)\hat{\beta}_{k;k'} c_{k;k'}(n) \frac{\sum_l \xi_{k',l} I_{k',1,l}(n)}{N_{k'}} . \tag{7}$$

With

$$I_{k,1,l}(n) = I_{k,1,1}(n-l+1)\pi_{k,l} = \Phi_k(n-l)\pi_{k,l} , \tag{8}$$

where $\pi_{k,l}$ is the probability that an infected person is still asymptomatic after l days, and

$$S_k(n) = S_k(0) - \sum_{n'=1}^{n} I_{k,1,1}(n') = S_k(0) - \sum_{n'=0}^{n-1} \Phi_k(n') \tag{9}$$

we obtain the recurrence relations

$$\Phi_k(n) = \left(\frac{S_k(0)}{N_k} - \sum_{n'=0}^{n-1} \frac{\Phi_k(n')}{N_k} \right) \sum_{k'} \beta(n)\hat{\beta}_{k;k'} c_{k;k'}(n) \sum_l \xi_{k',l} \pi_{k',l} \frac{\Phi_{k'}(n-l)}{N_{k'}} . \tag{10}$$

B.3 Effective reproduction numbers

To connect to effective reproduction numbers (when the population is not any more totally susceptible), we consider the number of new infections that will be generated in age group k by a newly infected person in age group k'. By neglecting changes during the short contagious period, we obtain from Eq. 10 the reproduction matrix

$$R_{k;k'}(n) = \frac{S_k(n)}{N_k} \beta(n)\hat{\beta}_{k;k'} c_{k;k'}(n) \frac{\sum_l \xi_{k',l} \pi_{k',l}}{N_{k'}} . \tag{11}$$

By considering the total number of new infections that will be generated by this infected person in group k', we obtain the group dependent reproduction numbers

$$R_{k'}(n) = \sum_k R_{k;k'}(n) . \tag{12}$$

Finally, with the new infected persons $\Phi_k(n)$ that are present at day n, we define the average reproduction number

$$R(n) = \sum_{k'} R_{k'}(n) \frac{\Phi_{k'}(n)}{\sum_{k'} \Phi_{k'}(n)} = \sum_{k,k'} R_{k;,k'}(n) \frac{\Phi_{k'}(n)}{\sum_{k'} \Phi_{k'}(n)} . \tag{13}$$

The pandemic grows for $R(n) > 1$ because the generated new infections $\sum_{k'} R_{k'}(n)\Phi_{k'}(n)$ is larger than the current value $\sum_{k'} \Phi_{k'}(n)$.

To characterize how the generated numbers of new infections will be distributed over the age groups, we define the reproduction distribution

$$RD_k(n) = \frac{\sum_{k'} R_{k;k'}(n)\Phi_{k'}(n)}{\sum_{k,k'} R_{k;k'}(n)\Phi_{k'}(n)}\,, \tag{14}$$

such that $\sum_k RD_k(n) = 1$. To characterize whether the infection grows or declines in age group k, we define the reproduction growth index

$$RGI_k(n) = \frac{\sum_{k'} R_{k;k'}(n)\Phi_{k'}(n)}{\Phi_k(n)} - 1\,. \tag{15}$$

The pandemic grows in group k for $RGI_k(n) > 0$.

B.4 Transition probabilities

The model probabilities $p_{k,h,j,l;i}(n_0)$ to transition from compartment j to compartment i (the label i also comprises the deceased and recovered compartment) are computed from the overall probabilities $\tilde{P}_{k,h,j;i}$ to transition from compartment j to compartment i, and the distributions $\tilde{p}_{k,h,j,l;i}(n_0)$ that specify at which day l this transition occurs. The time n_0 indicates that these probabilities might change at time n_0. We simplify the notation and omit the labels k, h and n_0 for the following calculations.

The probabilities $p_{j,l;i}$ satisfy the normalisation conditions $\sum_i p_{j,l;i} = 1$. In contrast, $\tilde{P}_{j;i}$ and $\tilde{p}_{j,l;i}$ satisfy the normalization conditions $\sum_{i\neq j} \tilde{P}_{j;i} = 1$ and $\sum_{l=1}^{l_{max}} \tilde{p}_{j,l;i} = 1$. The probability $\tilde{P}_{j;j}$ to remain in a compartment j is zero, since all infected will eventually recover or die. For numerical reasons, we truncate $\tilde{p}_{k,j,l;i}$ at a value l_{max} that is chosen sufficiently large. We chose $l_{max} = 60$ for $j = 3, 4$ and $l_{max} = 80$ for $j = 5$, since it takes longer for hospitalized to recover after ICU. As a function of $\tilde{P}_{j;i}$ and $\tilde{p}_{j,l;i}$ the probabilities $p_{j,l;i}$ can be computed as

$$p_{j,l;i} = \frac{\tilde{P}_{j;i}\tilde{p}_{j,l;i}}{1 - \sum_i \sum_{l'=1}^{l-1} \tilde{P}_{j;i}\tilde{p}_{j,l';i}} \quad (j \neq i)\,. \tag{16}$$

and $p_{j,l;j} = 1 - \sum_{i\neq j} p_{j,l;i}$. Because the probability to be found in compartment j after l_{max} days is zero, we have $p_{j,l_{max};j} = 0$.

B.5 Equations to compute the number of infected in a compartment as a function of influx and initial condition

We now derive matrix equations to compute the time evolution of the number of infected in a compartment j with a given initial condition and a prescribed influx. We need these expression to implement fitting procedures. The probability to be found in compartment j after l days is

$$\pi_{j,l} = \prod_{l'=1}^{l-1} p_{j,l';j}\,, \quad 1 \leq l \leq l_{max}\,. \tag{17}$$

The occupancy of compartment j at time n consists of persons that originate from an initial condition, and persons that joined this compartment via influx from other compartments.

B.5.1 Occupancy and fluxes to other compartments as a function of a given influx

The occupancy $O_j(n)$ due to the influx $I_{j,1}(n)$ from other compartments is given by the convolution

$$O_j(n) = \sum_{l=1}^{l_{max}} I_{j,l}(n) = \sum_{l=1}^{\min(n,l_{max})} I_{j,1}(n+1-l)\pi_{j,l}\,. \tag{18}$$

The number of persons $C_{j;i}(n)$ that change to a different compartment i is

$$C_{j;i}(n) = \sum_{l=1}^{l_{max}} I_{j,l}(n)p_{j,l;i} = \sum_{l=1}^{\min(n,l_{max})} I_{j,1}(n+1-l)\pi_{j,l}p_{j,l;i}\,. \tag{19}$$

The number of new persons in compartment i that originate from compartment j is $I_{i,1;j}(n+1) = C_{j;i}(n)$.

B.5.2 Occupancy and fluxes to other compartments as a function of an initial condition

We now consider the occupancy that originates from an initial condition $I_{j,l}(n_0)$ at day n_0 (without loss of generality we consider $n_0 = 1$). The number of infected at a future day n that are still in compartment j is

$$I_{j,l}(n) = I_{j,l-n+1}(1) \prod_{l'=l-n+1}^{l-1} p_{j,l';j}\,, \quad n \le l \le l_{max}\,. \tag{20}$$

The occupancy $\hat{O}_j(n)$ due to the initial condition therefore is

$$\hat{O}_j(n) = \sum_{l} I_{j,l}(n) = \sum_{l=1}^{l_{max}} M_{n,l} I_{j,l}(1) \tag{21}$$

where

$$M_{n,l} = \prod_{l'=l}^{l+n-2} p_{j,l';j} \tag{22}$$

For $n + l > l_{max} + 2$ we define $M_{n,l} = 0$.

The number of persons that change to a different compartment i is

$$\hat{C}_{j;i}(n) = \sum_{l} I_{j,l}(n)p_{j,l;i} = \sum_{l=1}^{l_{max}} M^i_{n,l} I_{j,l}(1) \tag{23}$$

with

$$M^i_{n,l} = p_{j,l+n-1;i} M_{n,l}\,. \tag{24}$$

For $l + n > l_{max} + 1$ we define $M^i_{n,l} = 0$.

B.6 Method to implement parameter changes

To implement parameter changes from P_{old} to P_{new} at day n_0 we use

$$P(n) = P_{old} + \frac{1}{2}\left(1 + \tanh\left(\frac{n - n_0}{\Delta}\right)\right)(P_{new} - P_{old}) \tag{25}$$

with $\Delta = 1$.

B.7 Discrete Gamma distribution

To fit distributions we use the discrete Gamma distribution defined as [2]

$$\tilde{p}_l = \frac{F(l) - F(l-1)}{F(l_{max})}\,, \quad l \leq 1 \leq l_{max}\,, \tag{26}$$

with

$$F(x) = \int_0^x \frac{1}{\Gamma(a)b}\left(\frac{y}{b}\right)^{a-1} e^{-\frac{y}{b}}\,dy\,. \tag{27}$$

The distribution depends on two parameters $a > 0$ and $b > 0$.

B.8 Fitting procedure

For the fittings we used the constrained optimization procedure *fminsearchbnd* from [3], which is based on MATLAB's *fminsearch* procedure. We use the matrix equations derived section B.5 to sequentially compute for each compartment the occupancy and outflux to other compartments as function of the initial condition and the influx from other compartments. For example, with the computed influx of new hospitalisations, we compute the future occupancies and fluxes between hospital compartments (as well as number of death and recovered) as a function the switching parameters. We then use data, simulation results and a cost function (here we chose the mean squared error) together with the constrained optimization procedure to fit the specified parameters.

References

[1] G. Béraud, S. Kazmercziak, P. Beutels, D. Levy-Bruhl, X. Lenne, N. Mielcarek, Y. Yazdanpanah, P.-Y. Boëlle, N. Hens, and B. Dervaux, "The french connection: The first large population-based contact survey in france relevant for the spread of infectious diseases," *PLOS ONE*, vol. 10, no. 7, pp. 1–22, 2015.

[2] Chakraborty, "Discrete gamma distributions: Properties and parameter estimations," *Communications in Statistics - Theory and Methods*, vol. 41, no. 18, pp. 3301–3324, 2012.

[3] J. D'Errico, "fminsearchbnd, fminsearchcon (https://www.mathworks.com/matlabcentral/fileexchange/8277-fminsearchbnd-fminsearchcon)," *MATLAB Central File Exchange*, 2021.
