# Supplementary figures and images for: Data-driven multiscale dynamical framework to control a pandemic evolution with non-pharmaceutical interventions
Source: PLoS One. 2023 Jan 17;18(1):e0278882. doi: 10.1371/journal.pone.0278882 (PMC9844884; doi:10.1371/journal.pone.0278882)

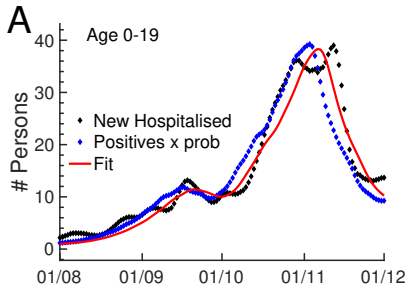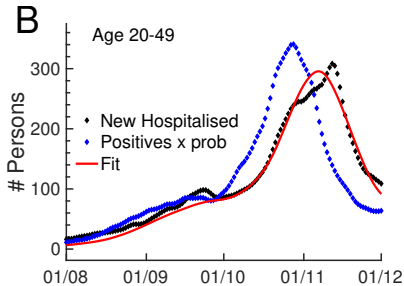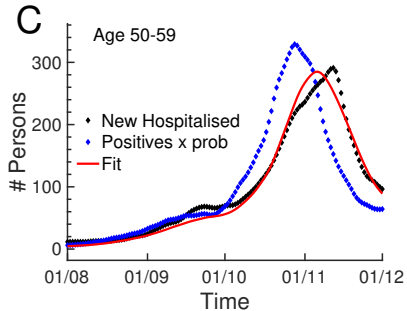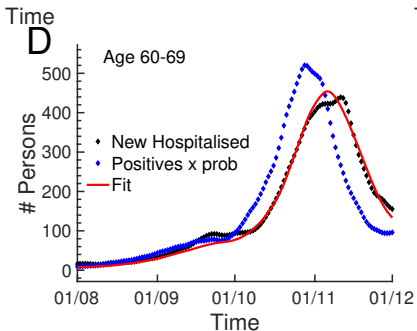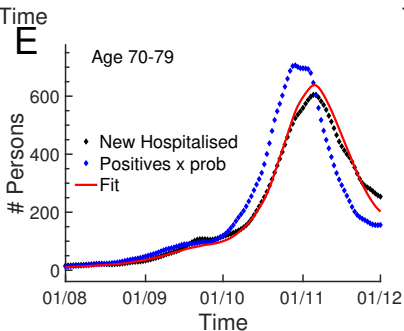

Supplement: S1 File — (ZIP) [file pone.0278882.s001.zip › SupportingInformation/figureSI_2.pdf]

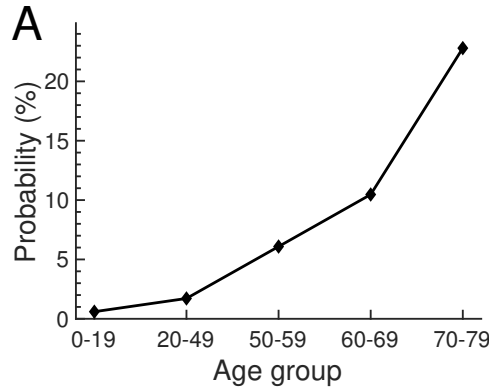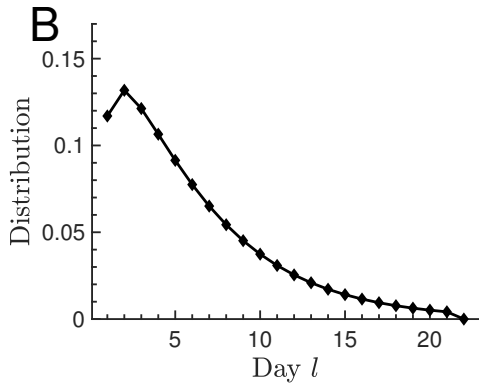

Supplement: S1 File — (ZIP) [file pone.0278882.s001.zip › SupportingInformation/figureSI_1.pdf]
